# Visualizing α‐synuclein pathology in live brain: A new platform for Parkinson's disease research

**DOI:** 10.1002/ctm2.70850

**Published:** 2026-10-01

**Authors:** Minhui Yu, Peng Cao

**Affiliations:** ^1^ Chinese Academy of Medical Sciences & Peking Union Medical College Beijing China; ^2^ National Institute of Biological Sciences Beijing China; ^3^ Tsinghua Institute of Multidisciplinary Biomedical Research Tsinghua University Beijing China

Parkinson's disease (PD) is a neurodegenerative disorder with rapidly rising prevalence and poses a significant global health concern in the coming decades.^1^ Motor deficits (e.g., resting tremor) and non‐motor features (e.g., gut motility dysfunction) together constitute its clinical picture.^2^, ^3^ At the core of its pathogenesis lies α‑synuclein (α‑Syn),^4^ a presynaptic protein encoded by the *Snca* gene.^5^ In its native conformation, α‐Syn remains largely unstructured, accumulates heavily at presynaptic boutons, and there governs the trafficking and fusion of synaptic vesicles.^6^ Upon misfolding and aggregation, however, α‐Syn forms Lewy bodies and Lewy neurites that progressively spread and inflict neuronal damage, and acts as a critical driver of disease progression.^7^, ^8^ Consequently, α‐Syn is considered a central target for both mechanistic studies and therapeutic development in PD. Despite this recognition, the field has long lacked technological tools capable of precisely and dynamically tracking α‐Syn aggregates in the live brain, severely constraining both mechanistic studies and the development of therapeutic interventions for PD. To address this unmet need, we introduce genetically encoded fluorescent reporters for labelling α‐Syn inclusions in neurons, including both *α‐Syn‐6H‐EGFP* and *α‐Syn‐6H‐tdT*, that enable direct visualization of α‐Syn aggregates and their spatiotemporal propagation in the live brain, thereby offering a powerful platform for studying PD pathogenesis and advancing therapeutic development.^9^

## 1 GENETICALLY ENCODED REPORTERS FOR VISUALIZING α‐SYN INCLUSIONS

To enable direct visualization of α‐Syn aggregation in the live brain, we designed a series of fusion proteins by fusing mouse α‐Syn to either EGFP or tdTomato (tdT) via peptide linkers. In cultured hippocampal neurons, we systematically screened seven linker variants and demonstrated that the 6‐histidine (6H)‐linked reporters exhibited the highest specificity and efficiency among all tested variants in labelling α‐Syn inclusions induced by preformed fibrils (PFFs). Based on this result, we identified the 6H linker as the optimal design and used it to construct two reporter genes: *α‐Syn‐6H‐EGFP* and *α‐Syn‐6H‐tdT*. Notably, both reporters exhibit a notable aggregation‐dependent fluorescence enhancement: when α‐Syn exists as monomers, the fluorescence signal remains weak; however, upon integration into aggregates, the fluorescence intensity increases approximately five‐fold. Moreover, the reporters achieve a co‐localization rate of 93%–97% with phosphorylated α‐Syn (p‐α‐Syn) inclusions, the gold‐standard marker of authentic Lewy pathology,^10^ placing their detection accuracy at the forefront of current technologies.

The reporter system also demonstrates excellent biocompatibility. Through detergent extraction assays using 1% Triton X‐100, we found that non‐aggregated *α‐Syn‐6H‐tdT* and *α‐Syn‐6H‐EGFP* remained soluble, whereas reporter‐labelled inclusions were detergent insoluble, confirming their stable integration into α‐Syn aggregates. Furthermore, in *Snca‐/‐* neurons lacking endogenous α‐Syn, PFFs treatment induced p‐α‐Syn+ inclusions in less than 1% of neurons expressing the reporters, indicating that these fusion proteins have a markedly lower aggregation propensity than α‐Syn itself and do not initiate aggregation on their own. Expression of the reporters also did not alter the density of PFF‐induced p‐α‐Syn+ inclusions in wild‐type neurons, demonstrating that they do not interfere with the normal physiological function of endogenous α‐Syn.

To extend these findings to in vivo settings, we also designed reporter mouse lines. Using CRISPR/Cas9 technology, we generated *Snca‐6H‐tdT* and *Snca‐6H‐EGFP* knock‐in (KI) mouse lines, in which reporter expression faithfully recapitulates the endogenous α‐Syn expression pattern, as confirmed by immunoblotting and immunohistochemical analyses showing preferential expression in the forebrain and presynaptic localization in the hippocampal CA3 region. Subsequently, injection of α‐Syn PFFs into various brain regions, including the cortical (motor cortex), subcortical (hippocampus, dorsal striatum), and brainstem regions such as the substantia nigra pars compacta (SNc), and dorsal motor nucleus of the vagus (DMV), demonstrated that these reporters reliably label p‐α‐Syn+ inclusions across multiple brain areas. Importantly, uninjected reporter mice showed no such inclusions, indicating that the reporters themselves do not drive endogenous α‐Syn aggregation. In addition, through the generation of Cre‐dependent mouse lines (*RCL‐Snca‐6H‐EGFP*), these reporters enable selective labelling of α‐Syn inclusions in specific neuronal subtypes, including dopaminergic, glutamatergic and cholinergic neurons. This cell‐type‐specific approach allows for the investigation of α‐Syn inclusion‐induced pathological alterations in both transcriptomes and metabolomes at single‐cell resolution. These mouse lines collectively offer a versatile in‑vivo platform for studying α‑Syn aggregate biology.

## 2 VERSATILE APPLICATIONS OF THE REPORTER MOUSE LINES

By combining these reporters with advanced imaging, electrophysiology, and single‐cell omics, we have uncovered three major insights into α‐Syn pathology (Figure 1).

**FIGURE 1:**
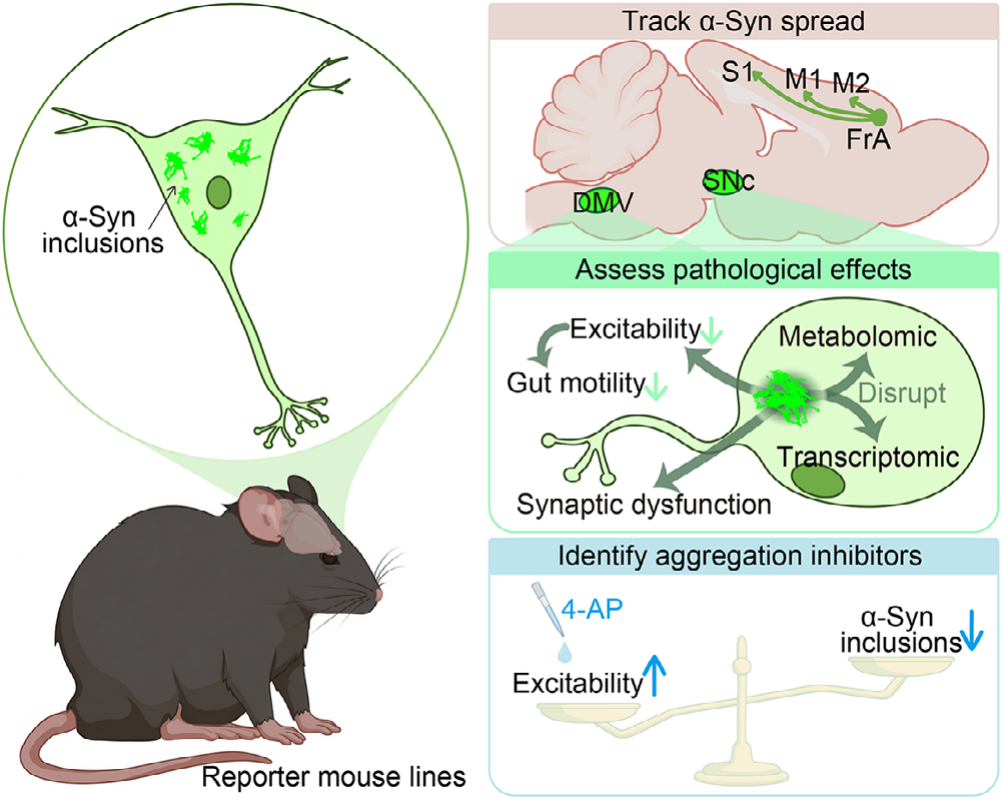
Schematic overview of the applications of the reporter mouse lines.

First, we addressed the question of aggregate propagation. After injecting α‑Syn PFFs into the frontal association cortex (FrA) of Snca‑6H‑EGFP KI mice, we used two‑photon imaging in awake animals to monitor the dissemination of inclusions from the FrA to primary motor, secondary motor, and primary somatosensory cortices over 84 days. This allowed us to directly visualize the dissemination process in vivo, offering strong support for the prion‑like spread hypothesis largely inferred from post‐mortem studies. The ability to monitor the same animal over time opens valuable opportunities for understanding PD pathogenesis and evaluating therapeutic interventions.

Second, to explore how α‑Syn inclusions exert their pathological effects, we employed a multi‐faceted approach combining in vivo calcium imaging, whole‐cell patch‐clamp, single‐cell patch‐seq transcriptomics, and single‐cell mass spectrometry. These integrated analyses revealed that α‐Syn inclusions suppress spontaneous neuronal activity, impair both excitatory and inhibitory synaptic transmission, and disrupt a broad range of cellular pathways, including mitochondrial function, the ubiquitin‐proteasome system, lysosomal‐endosomal trafficking, calcium homeostasis, and neuronal metabolism. To bridge these cellular deficits to a behaviourally relevant circuit, we focused on cholinergic neurons in the DMV, a brain region critical for gastrointestinal control.^11^ In this circuit, α‐Syn inclusions reduced neuronal excitability and directly caused gut motility dysfunction, mirroring one of the earliest and most common non‐motor symptoms of PD.^12^ This observation provides evidence for a direct causal chain from molecular pathology to a clinically relevant phenotype, offering a new entry point for understanding and treating the prodromal phase of PD. Collectively, our findings provide a comprehensive mechanistic framework for how α‐Syn inclusions drive neuronal dysfunction at both the cellular and circuit levels, highlighting the power of cell‐type‐specific approaches in dissecting the heterogeneity of PD pathology.

Third, we leveraged the reporter system as a live‐cell screening platform to identify inhibitors of α‐Syn inclusion formation. This system enabled us to validate the efficacy of known aggregation inhibitors, such as heparin and squalamine, and more importantly, led to the discovery that 4‐aminopyridine (4‐AP), a potassium channel inhibitor used clinically for multiple sclerosis,^13^ significantly suppresses α‐Syn inclusion formation by enhancing neuronal action potential firing. Mechanistically, this effect revealed a previously unrecognized bidirectional relationship between neuronal activity and α‐Syn aggregation. In vivo, oral administration of 4‐AP reduced α‐Syn pathology in both the SNc and the DMV, and concurrently ameliorated both motor and non‐motor (gastrointestinal) deficits in PD mouse models. Collectively, these findings not only identify a novel therapeutic target and a promising lead compound for PD, but also highlight the translational power of the reporter system as a versatile platform for drug discovery.

## 3 CLINICAL CHALLENGES AND FUTURE PERSPECTIVES

Our genetically encoded fluorescent reporters feature high specificity, high sensitivity, and excellent biocompatibility, making the system suitable not only for PD but also for other α‑synucleinopathies, such as dementia with Lewy bodies and multiple system atrophy.

This reporter system, together with its accompanying mouse lines, is now openly available through our laboratory and constitutes a versatile and powerful platform for studying α‐Syn pathology in the live brain. The aggregation‐dependent fluorescence enhancement offers a quantitative readout of pathology burden, which in turn facilitates rapid and cost‐effective identification of compounds that modulate α‐Syn inclusion formation, with broad applications in biomarker discovery and high‑content drug screening. The cell‐type‐specific labelling capacity allows us to probe how pathology differentially impacts vulnerable neuronal subsets, which may help prioritize candidate therapeutic targets based on subtype vulnerability. Moreover, the ability to track α‐Syn inclusion progression in the same animal over time provides a powerful preclinical platform for evaluating candidate therapies. Overall, we see this system as a versatile resource for both mechanistic and translational research in α‑synucleinopathies.

## AUTHOR CONTRIBUTIONS

All authors contributed to the writing of this editorial.

## CONFLICT OF INTEREST STATEMENT

The authors declare no conflicts of interest.
